# Supplementary material for: Impact of Peptidoglycan Recycling Blockade and Expression of Horizontally Acquired β-Lactamases on Pseudomonas aeruginosa Virulence
Source: Microbiol Spectr. 2022 Feb 16;10(1):e02019-21. doi: 10.1128/spectrum.02019-21 (PMC8849096; doi:10.1128/spectrum.02019-21)
Supplement: SUPPLEMENTAL FILE 1 — Supplemental material. Download SPECTRUM02019-21_Supp_1_seq4.pdf, PDF file, 0.8 MB [file spectrum02019-21_supp_1_seq4.pdf]

Supplemental Material

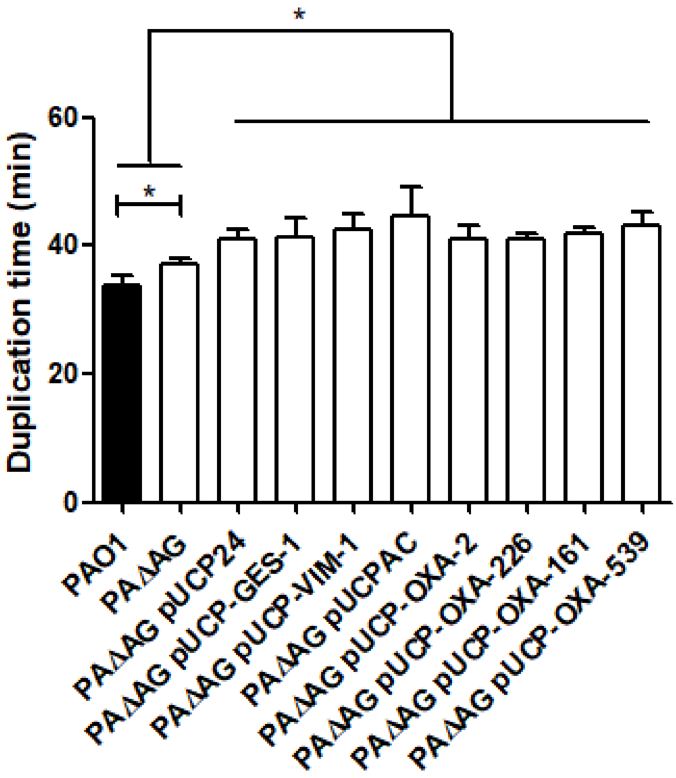

FIG S1. Duplication times (minutes) in LB broth of exponentially-growing cells of *P. aeruginosa* strains and derivatives harboring different cloned β-lactamases. Columns represent mean values plus SDs (error bars) from three independent experiments. The values that are significantly different ($P < 0.05$ in Tukey's post-hoc test after ANOVA) are depicted by horizontal capped lines and asterisks. Strains have been grouped with horizontal uncapped lines in order to lighten up the figure when displaying statistical significance.

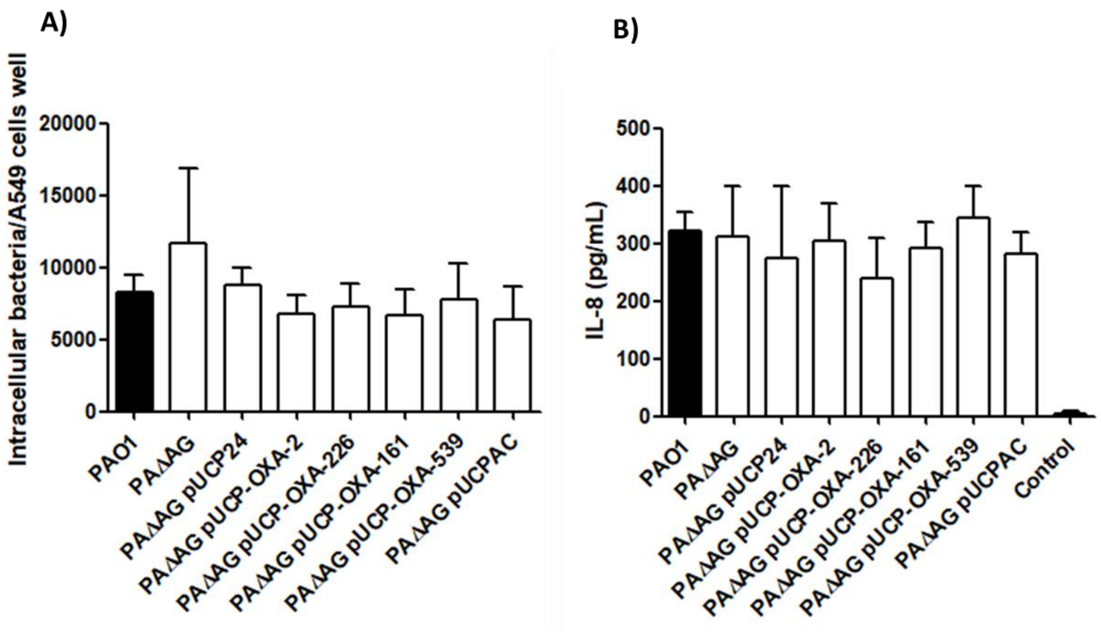

FIG S2. Cell culture experiments with PAO1 and AmpG-defective derivatives harboring the different cloned β-lactamases. All values represented correspond to the means (columns) plus SDs (error bars) obtained from at least three wells from three independent 24-well plates (all data are shown on a linear scale). **A)** Invasion assay. The number of invasive CFUs per well of A549 cells after infection is shown. **B)** IL-8 response of A549 cells (released interleukin in pg/mL) after infection with the indicated strain. Mean released IL-8 from uninfected wells is also shown for comparison.

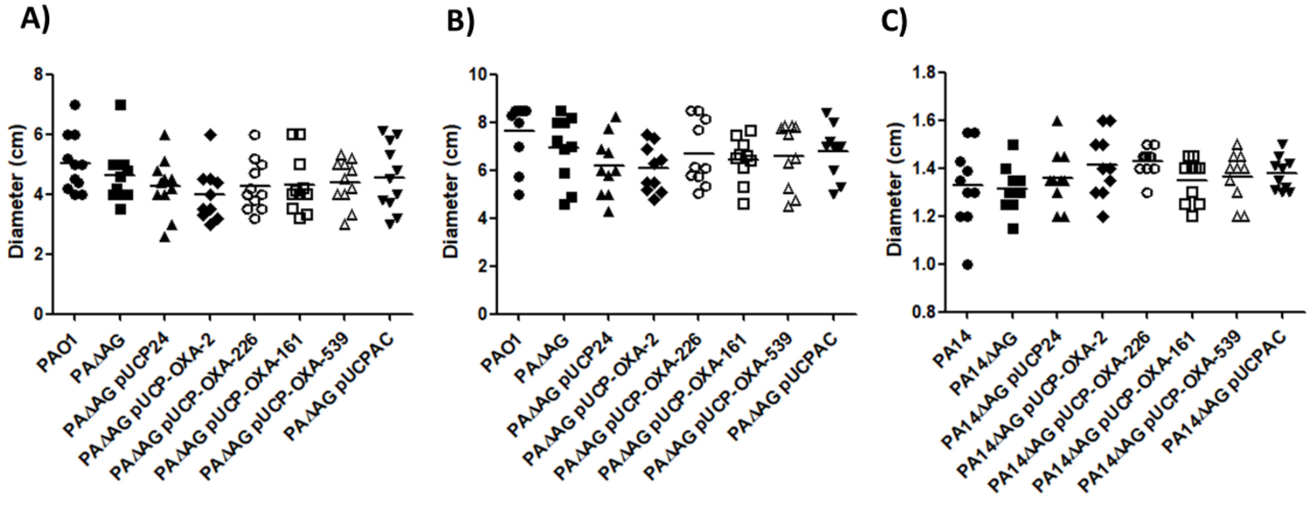

FIG S3. Motility of *P. aeruginosa* strains (wildtype and PAΔAG mutant) harboring the different cloned β-lactamases. The diameters (cm) of the motility areas of 10 different inoculations per strain were measured and plotted, and are depicted by each individual symbol. Mean values for the 10 measures are indicated by short black bars. A) Swimming motility of PAO1 and derived strains. B) Swarming motility of PAO1 and derived strains. C) Twitching motility of PA14 and derived strains.

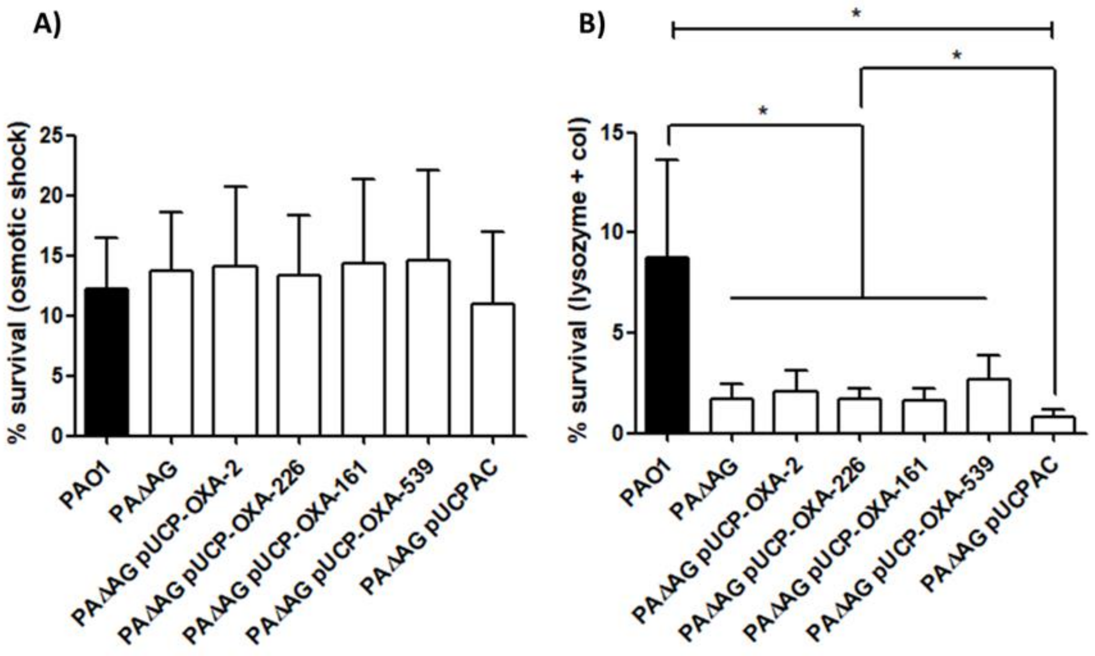

FIG S4. Susceptibility of PAO1 and PAΔAG strains harboring the different cloned β-lactamases to physical/lytic aggressions against the peptidoglycan. Treatments were performed as explained in the Materials and Methods section, and while the columns represent the mean values of at least three independent determinations per strain, the error bars correspond to SDs. All data are displayed on a linear scale. Strains have been grouped with an horizontal uncapped line when no statistical difference existed between them, in order to lighten up the figure. **A)** Percentage of bacterial survival (with regard to the initial inoculum) after hypo-osmotic shock treatment: bacteria suspended for 24h in double distilled water at room temperature and gentle agitation. **B)** Percentage of bacterial survival (with regard to the initial inoculum) after treatment with chicken egg white lysozyme (25 mg/L, 1 h at 37 °C and 180 rpm agitation, with 0.025 mg/L of colistin acting as a permeabilizer). There were no statistically significant differences among the strains regarding their susceptibility to the treatments of lysozyme alone or colistin alone (data not shown).
